# Strategies for Developing Transition Metal Phosphides in Electrochemical Water Splitting

**DOI:** 10.3389/fchem.2021.700020

**Published:** 2021-11-03

**Authors:** Jie Ying, Huan Wang

**Affiliations:** School of Chemical Engineering and Technology, Sun Yat-sen University, Zhuhai, China

**Keywords:** transition metal phosphides, hydrogen evolution reaction, oxygen evolution reaction, water splitting, modulated strategies

## Abstract

Electrochemical water splitting involving hydrogen evolution reaction (HER) and oxygen evolution reaction (OER) is a greatly promising technology to generate sustainable and renewable energy resources, which relies on the exploration regarding the design of electrocatalysts with high efficiency, high stability, and low cost. Transition metal phosphides (TMPs), as nonprecious metallic electrocatalysts, have been extensively investigated and proved to be high-efficient electrocatalysts in both HER and OER. In this minireview, a general overview of recent progress in developing high-performance TMP electrocatalysts for electrochemical water splitting has been presented. Design strategies including composition engineering by element doping, hybridization, and tuning the molar ratio, structure engineering by porous structures, nanoarray structures, and amorphous structures, and surface/interface engineering by tuning surface wetting states, facet control, and novel substrate are summarized. Key scientific problems and prospective research directions are also briefly discussed.

## Introduction

**FIGURE 1:**
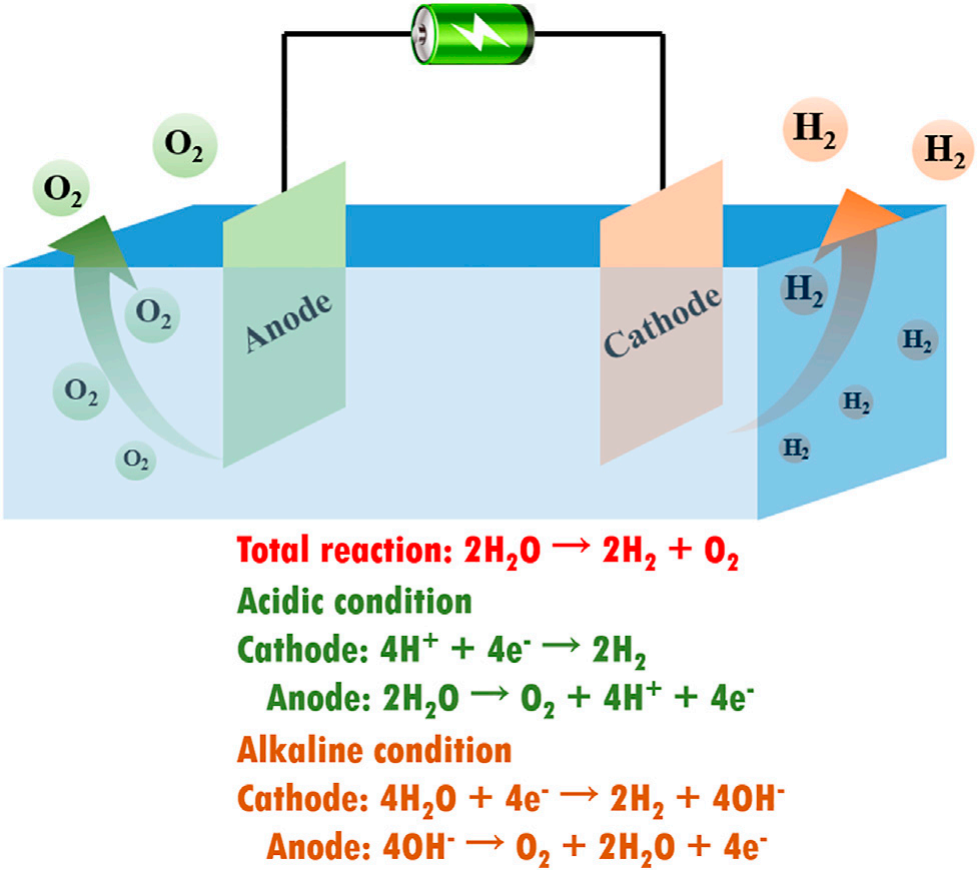
Schematic illustration of an electrolyzer for electrochemical water splitting and corresponding reaction equations in different reaction conditions.

In the past decades, global energy consumption has been growing dramatically, with fossil fuels still providing over 80% of energy consumption, resulting in severe energy crisis and greenhouse effect (Zhang et al., 2017a; Peng et al., 2016). To address the key issue of these energy sources, researchers have begun to exploit clean and renewable energy resources such as solar energy, geothermal energy, wind power, and hydropower (Gallagher et al., 2015; Hou et al., 2011). Since hydrogen is regarded as a pollution-free energy source with ultrahigh energy density, water electrolysis has attracted tremendous attention for producing hydrogen energy from water in abundance (Peng et al., 2014; Ying et al., 2017; Yu et al., 2019a). As shown in Figure 1, electrochemical water splitting consists of two half-cell reactions, namely, hydrogen evolution reaction (HER) at the cathode and oxygen evolution reaction (OER) at the anode. The current state-of-the-art water electrolysis technology requires the use of precious metal (e.g., Pt and IrO_2_) electrocatalysts (Fujishima et al., 2008; Stoerzinger et al., 2015). Nevertheless, the high cost and low abundance of precious metals are restricting the widespread application of water electrolysis technology (Ying et al., 2014a; Ying et al., 2018a; Xiao et al., 2019; Xiao et al., 2021a).

Accordingly, tremendous substantial efforts have been devoted to the development of sustainable alternative electrocatalysts, one of which involves high efficiency, high stability, and low cost. Thus, many nonprecious metal electrocatalysts including carbon/carbon-based nanomaterials (Lu et al., 2015; Han et al., 2019; Ji et al., 2019), metal chalcogenides (Li et al., 2016), carbides (Yang et al., 2019; Lai et al., 2020), borides (Li et al., 2019), nitrides (Ye et al., 2018; Tareen et al., 2019), oxides (Song et al., 2018; Li et al., 2020), and especially phosphides (Ren et al., 2020; Sarkar et al., 2020) are currently representative electrocatalytic materials for both HER and OER. Among them, transition metal phosphides (TMPs) have been widely investigated and demonstrated to be very suitable for electrochemical water splitting (Dutta and Pradhan, 2017; Wang et al., 2018).

As the first nickel phosphide was prepared for vapor phase catalysis in the 1950s, it has been gradually depleted for a long time (Sweeny et al., 1958). In the 1990s, Kupka et al. first used metal phosphides as electrocatalysts (Kupka and Budniok, 1990). In 2005, Liu et al. presented the high HER activity of Ni_2_P(001) facet by density functional theory (DFT) calculations and suggested that the Ni-P bonds form a weak “ligand effect” that endows the fast dissociation of thiophene and hydrogen (Liu and Rodriguez, 2005). In 2013, Lewis et al. used nanostructured TMPs as HER electrocatalysts in acid media (Popczun et al., 2013). Until 2015, Yoo et al. made further progress demonstrating that the surface oxidized compounds are the true catalytic site of the metal phosphides (Ryu et al., 2015). Inspired by this work, massive research studies on TMPs for water electrolysis in the past several years have been reported. For example, Liu et al. reported an oxygen doping strategy to prepare an effective NiCoP electrocatalyst with optimized hydrogen adsorption energy and plentiful exposed active sites (Liu et al., 2018a).

Currently, the field of synthesis of TMP electrocatalysts for water splitting is experiencing a prosperous development with increasing achievements. It is necessary to timely provide a brief overview of this type of advanced material. In this minireview, we provide a general overview of the recent advances in efficient TMPs for electrochemical water splitting based on the understanding of their relationship between structure and performance. The developments in the design strategies based on composition engineering, structure engineering, and surface/interface engineering are summarized. Moreover, key scientific problems and prospective research directions are also proposed.

### Composition Engineering

Considering the influence of electron structure and intermediate adsorption energy, the introduction of elements into TMPs, such as element doping, hybridization with other compositions, and tuning their molar ratios is often used to improve their electrocatalytic performance.

### Element Doping

**FIGURE 2:**
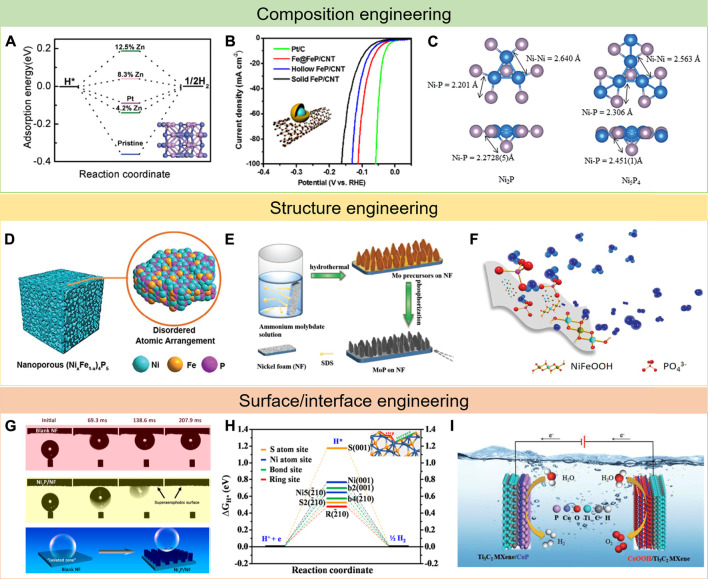
Typical examples of strategies for the development of TMP electrocatalysts: **(A–C)** composition engineering, reproduced with permission from ref. Liu et al. (2017), Li et al. (2017), and Laursen et al. (2015), respectively; **(D–F)** structure engineering, reproduced with permission from ref. Xu et al. (2018a), Jiang et al. (2018), and Hu et al. (2017), respectively; and **(G–I)** surface/interface engineering, reproduced with permission from ref.(Yu et al. (2019b), Feng et al. (2015), and Yan et al. (2020), respectively.

In general, doping foreign elements can boost the intrinsic activity of electrocatalysts (Niu et al., 2019; Song et al., 2021; Gao et al., 2019). What is more, the optimal ratio of doping elements can be predicted in advance *via* DFT calculations (Peng et al., 2021; Fu et al., 2019). Hence, the electrocatalytic behavior can be precisely regulated at the atomic scale. Most single metal phosphides have a limited intrinsic activity due to the difficulty in balancing the adsorption and desorption of reaction intermediates (Parra-Puerto et al., 2019; Read et al., 2016; Dutta et al., 2016). To solve the shortcomings, incorporating foreign atoms into the single TMPs has been studied by many research groups (Wu et al., 2018; Du et al., 2020; Yue et al., 2019; Shin et al., 2020; Lv et al., 2020). Liu et al. reported that doping element Zn into pristine CoP could significantly enhance HER performance (Liu et al., 2017). Because Zn had lower electron negativity as compared with Co, it could provide some electrons to nearby P atoms and generate some electron-deficient cations. As a result, the surrounding Co will lose more electrons, thus weakening H chemisorption strength with Co and improving HER performance (Figure 2A). Not only that many research studies have been achieved to explore the effects of introducing other elements such as N, S, or O into TMPs (Chang et al., 2018; Xi et al., 2018; Wang et al., 2019; Mu et al., 2021).

### Hybridization

Hybridization with other compositions also remarkably increased catalytic activity because of the strong synergistic effect between multiple compositions and the improved mass transportation ability. In a multicomponent composite, the contacted components/phases show special interactions (Wang et al., 2018). By the rational design of the heterogeneous structure, the physicochemical properties of the interface can be obviously changed, thus presenting better performance than the single bulk phase (He et al., 2018; Saha et al., 2020). Li et al. developed a novel Fe@FeP core–shell nanoparticles on carbon nanotubes as an efficient HER catalyst (Li et al., 2017). The resulting Fe@FeP hybrid catalyst presented a low overpotential of 53 mV at a current density of 10 mA cm^−2^ and a Tafel slope of 55 mV dec^−1^ due to the presence of strong electron interactions and synergistic effects between Fe and FeP (Figure 2B). DFT calculations showed that the hydrogen adsorption energy on Fe@FeP is very close to that on Pt (111), revealing the enhancement effect of the Fe@FeP core–shell structure. In addition, Yu et al. synthesized a FeP/Ni_2_P hybrid catalyst *via* the chemical vapor deposition method (Yu et al., 2018). The obtained hybrid catalyst showed excellent catalytic performance in HER because it preferentially exposed the most active facets compared to the FeP(001) crystal, which contributed to the high activity not observed in typical FeP crystals.

### Tuning the Molar Ratio of M/P

Since the first use of TMPs in electrocatalysis, enormous efforts have been devoted to pursuing the optimal metal/phosphorus (M/P) molar ratios (Callejas et al., 2015). As studied before, different M/P molar ratios lead to variations in crystal structures, hence directly influencing the electrocatalytic properties (Pei et al., 2017). In some situations, tuning molar ratios of M/P could boost excellent enhancements on HER activity. For example, Laursen et al. have explored the superior HER activity of Ni_5_P_4_ nanoparticles by researching the different catalytic performances between Ni_2_P and Ni_5_P_4_ nanoparticles (Laursen et al., 2015). The combination of the Volmer–Tafel mechanism and DFT calculation disclosed that Ni_5_P_4_ displayed a faster reaction rate because of the increased binding energy of the first hydrogenic intermediate, which in turn increases the second proton affinity. Moreover, the bond length of Ni-P in Ni_4_P_5_ is also longer than that in Ni_2_P (Figure 2C), explaining that Ni_4_P_5_ exhibited much better catalytic activity compared to Ni_2_P.

### Structure Engineering

Besides the composition engineering mentioned above, structure engineering is also an indispensable strategy to improve the water splitting performance of electrocatalysts. Three main aspects including porous structures, nanoarray structures, and amorphous structures are discussed in this section.

### Porous Structures

It is well known that the electrocatalytic reactions proceed on the surface of electrocatalysts; tiny pore structure could lead to improving the surface area and exposing more active sites (Peng et al., 2018; Xiao et al., 2021b; Ying et al., 2018b; Ying et al., 2014b). Since Erlebacher’s group put forward a continuum model to explain the fundamental mechanism of nanoporosity formation in the dealloying process (Erlebacher et al., 2001), many scientists have continuously continued to explore this. In this regard, Xu et al. developed a facile technique to prepare a nanoporous (Ni_x_Fe_1-x_)_4_P_5_ with controllable metal ratio (Figure 2D), which serves as a bifunctional catalyst for both HER and OER with an outstanding catalyst performance (Xu et al., 2018a). Sun et al. designed porous and multishelled Ni_2_P hollow spheres by using the carbon spheres as the template, subsequently phosphating along with thermal treatment (Sun et al., 2017). The hollow porous multishells and nanosized subunits endow Ni_2_P with short charge transport distances, abundant active sites, and high stability against agglomeration, representing outstanding OER catalytic performance.

### Nanoarray Structures

The nanoarray-structured self-supported electrodes, growing on the free-standing substrates, with discontinuous phase contact areas are very attractive due to avoiding the negative effects of binders and generating excellent stability (Liang et al., 2016; Zhang et al., 2020; Hou et al., 2020). In contrast to swarming bubbles adsorbed onto the planar surface of bulk materials, these nanoarrays can always show very preeminent electrocatalytic activity. For example, Shen et al. reported a porous MoP nanoflake array grown on nickel foam (MoP/NF) with excellent performance in water electrolysis (Figure 2E) (Jiang et al., 2018). In contrast to MoP, the enhanced electrocatalytic performance of MoP/NF is ascribed to the reduced size and the special nanoflake array structure. Moreover, besides avoiding the use of binders, the porous MoP/NF could be directly treated as both current collector and electrocatalyst, which made the active sites fully exposed and conductive to gas penetration and mass transfer. For another example, Su et al. successfully prepared self-supported NiMoP_2_ with grain boundary rich nanowire architecture on a carbon cloth substrate, showing small overpotential and remarkable electrochemical durability for water splitting (Wang et al., 2017). Such impressive characteristics can be tracked to the hierarchical architecture of the NiMoP_2_ nanowire in situ grown on a 3D carbon cloth substrate weakening the disintegration tendency of catalyst, and the special grain boundary nanowire structure of NiMoP_2_ provides the maximum number of electroactive surface/active sites.

### Amorphous Structures

Since vacancies and defects are often considered as active sites of catalysis, amorphous catalysts are widely researched because of the disordered domains containing lots of vacancies and defects (Yan et al., 2017; Chu et al., 2020). In general, amorphous structures are always rigorous to preparation conditions (Zhang et al., 2016; Anantharaj et al., 2017; Ray et al., 2017). For instance, Xiong et al. reported a high-efficiency OER electrocatalyst based on bulk amorphous NiFeP in both alkaline and acidic electrolytes (Hu et al., 2017). In this case, the element P with proper electronegativity has been utilized to stabilize Ni and Fe atoms as an amorphous metallic phase with high conductivity and may also form active species for enhancing OER performance. Besides, the metallic bonds facilitated the electron transfer, whereas the P atoms supplied suitable bonds with reaction intermediates (Figure 2F). At the same time, the coordination numbers of Ni and Fe in Ni_40_Fe_40_P_20_ were largely lower than those counterparts, indicating metal atoms in the Ni_40_Fe_40_P_20_ were unsaturated. Combining these features together, this Ni_40_Fe_40_P_20_ electrode manifested an outstanding performance in OER. Therefore, the amorphous NiFeP accomplishes unprecedentedly excellent OER performance.

### Surface/Interface Engineering

Surface/interface engineering, including the tuning surface wetting states, facet control, and novel substrate, is another effective way to enhance the performance of electrocatalysts. Since both mass transfer and gas delivery play a crucial role during water splitting, modifying the surface wettability such as superhydrophilicity is beneficial to HER and OER. In addition, the facet control and novel substrate are also beneficial to electrocatalytic properties *via* exposing more catalytic active sites and facilitating the electrolyte transfer.

### Tuning Surface Wetting States

During the electrolysis of water, the surface wettability such as superhydrophilicity and superaerophobicity of the electrocatalysts are key to the electrocatalytic process due to the evolution of gas bubbles in the solution (Hao et al., 2016; Tian et al., 2020). It is commonly thought that superhydrophilicity is advantageous for mass transfer inside the electrode and can improve the interaction between electrocatalysts and electrolytes (Kim et al., 2021; Riyajuddin et al., 2021). Likewise, the superaerophobicity can be beneficial to electrocatalysts by avoiding bubble effects (Sheng et al., 2020). The formed gas bubbles would adhere to the surface of the electrocatalyst during the water splitting process, which would be a barrier to the solution diffusing to the active sites and result in a dissatisfied performance (Xu et al., 2018b). Precise surface design can improve the surface’s superhydrophilicity and superaerophobicity to boost the interaction between electrodes and electrolytes and also solve the bubble adsorption issue during the electrocatalytic process. For example, Yu et al. prepare a Ni_2_P nanoarray catalyst with a unique superaerophobic surface feature grown on a Ni foam substrate, which represents remarkable electrocatalytic activity and stability in basic media (Yu et al., 2019b). The special superaerophobicity endows an outstanding capability to withstand inside and outside forces and releases the *in situ*–formed H_2_ bubbles timely (Figure 2G), resulting in highly efficient electrocatalytic activity and outstanding stability of the Ni_2_P/NF.

### Facet Control

While researching the composition of a nanostructure to TMPs that display reasonable electrocatalytic performance during water splitting progress, a large portion of research studies have focused on enhancing the intrinsic activity of active sites on the surface of nanostructures (Dutta et al., 2016; Lei et al., 2018; Ying et al., 2018c). Hence, there have been enormous efforts to prepare facet-controlled TMP nanostructures restricted by special facets which exert excellent catalytic activity (Liu and Rodriguez, 2005; Popczun et al., 2013). For example, Zhang et al. reported the ultrathin CoP nanosheets with dominant active facets by a chemical transformation method (Zhang et al., 2017b). The as-prepared samples consist of porous nanosheets, which are in single crystalline structure with a preferential [100] orientation. DFT calculation disclosed that on CoP (Lv et al., 2021) facets, a near-zero Gibbs free energy could lead to high utilization efficiency of active sites, therefore endowing (Lv et al., 2021) highly active CoP. In addition to single crystalline, other crystallines are, of course, affected by the crystal surface. For instance, Feng et al. reported novel {2—10} facet-exposed Ni_3_S_2_ nanosheet arrays on nickel foam (NF) (Figure 2H), which can efficiently catalyze both HER and OER (Feng et al., 2015). The excellent electrocatalytic performance in water splitting can be attributed mainly to the synergistic effect between the nanosheet array architecture and exposed {2—10} high-index facets.

### Novel Substrate

Until now, two novel substrates with good conductivity, high mechanical strength, and corrosion resistance in electrocatalysis fields have been developed, which can act as supporting materials for stabilizing electrocatalysts and facilitate the electron transfer from the external circuit to electrocatalysts (Liu et al., 2018b; Gao et al., 2021; Yu et al., 2020; Xiu et al., 2018). One of them is black phosphorus (BP), a kind of 2D material, which is well known for its intriguing physicochemical properties, such as high charge–carrier mobility, tunable bandgap, and highly anisotropic characters (Fei and Yang, 2014; Jiang and Park, 2014; Liu et al., 2014). In view of these merits of BP, Wu et al. designed novel Ni_3_N-Ni_2_P-BP heterostructure nanosheets with enhanced OER activity in alkaline conditions (Wu et al., 2019). The DFT calculation demonstrates that Ni_2_P is very close to the ideal value compared to the Gibbs free energies of Ni_3_N and BP. In addition, the electron transfer rate can be improved by the metallic nature of the Ni_3_N-Ni_2_P-BP catalyst. As a result, the heterostructure of Ni_3_N-Ni_2_P-BP catalysts can remarkably enhance the OER activity. As another promising 2D material, transition metal carbides and nitrides (known as MXenes) with the features of great mechanical stability, high conductivity, and wide chemical variety display great potential as substrates (Du et al., 2018; Yoon et al., 2019; Lv et al., 2021). For example, Yan et al. successfully prepared novel CoP nanosheet arrays on the surface of Ti_3_C_2_ MXene nanosheets (CoP/Ti_3_C_2_ MXene) (Figure 2I) (Yan et al., 2020). Benefiting from the unique structure and synergistic effect between the active CoP and Ti_3_C_2_ MXene nanosheets, the 3D MXene matrix not only prevents the self-aggregation of active sites but also significantly facilitates the electrolyte accessibility and enhances the charge/mass transfer. Hence, the CoP/Ti_3_C_2_ MXene heterostructure represents a largely improved electrocatalytic activity and remarkable stability in HER over a wide pH range.

## Conclusion and Outlook

This review has summarized the reported strategies for developing TMP electrocatalysts in electrochemical water splitting, including composition engineering, structure engineering, and surface/interface engineering. These strategies can be utilized as common and efficient strategies for preparing high-performance electrocatalysts. Moreover, the strategies discussed above can be modified and/or extended to other systems although we only focused on a limited number of examples.

Although rapid and significant development has been made in the synthesis of TMP electrocatalysts with superior HER and/or OER performance, the research in this field is still at the exploration stage, and several issues need to be addressed, such as preparation of TMPs with a special facet, stabilization of TMPs in acidic OER, and surface oxidation of TMPs. To better understand the in-depth reason for the enhanced electrocatalytic performance, two research aspects are recommended: 1) *in situ* structural characterization for investigating the catalytic active sites and 2) theoretical reaction simulation for predicting the optimized structures/compositions. Combining these two aspects, the field of TMP electrocatalysts for water splitting will undoubtedly keep moving forward rapidly.
